# Endothelial Barrier Disruption by Lipid Emulsions Containing a High Amount of N3 Fatty Acids (Omegaven) but Not N6 Fatty Acids (Intralipid)

**DOI:** 10.3390/cells11142202

**Published:** 2022-07-14

**Authors:** Emilie Gueguen, Yasser Morsy, Michael Scharl, Stefanie D. Krämer, Michael Zaugg, Martin Hersberger, Gerhard Rogler, Marcin Wawrzyniak

**Affiliations:** 1 Faculty of Fundamental and Applied Sciences, University of Poitiers, 86000 Poitiers, France; emilie.gueguen@usz.ch; 2 Department of Gastroenterology and Hepatology, University Hospital Zurich, University of Zurich, 8091 Zurich, Switzerland; yasser.morsy@usz.ch (Y.M.); michael.scharl@usz.ch (M.S.); gerhard.rogler@usz.ch (G.R.); 3 Institute of Pharmaceutical Sciences, Department of Chemistry and Applied Biosciences, ETH Zurich, 8039 Zurich, Switzerland; stefanie.kraemer@pharma.ethz.ch; 4 Department of Anesthesiology and Pain Medicine and Cardiovascular Research Centre, University of Alberta, Edmonton, AB T6G 2R3, Canada; mzaugg@ualberta.ca; 5 Division of Clinical Chemistry and Biochemistry, University Children’s Hospital Zurich, 8032 Zurich, Switzerland; martin.hersberger@kispi.uzh.ch

**Keywords:** parenteral nutrition, endothelial cells, endothelial barrier, lipid emulsions

## Abstract

Lipid emulsions are crucial for life-saving total parenteral nutrition (TPN). Their composition provides a high amount of essential fatty acids and calories for millions of patients with serious diseases. Nevertheless, several TPN-mediated side-effects have been reported in over 90% of patients. This project aimed to investigate the effect of a high amount of ω3 fatty acids (Omegaven^®^) emulsion vs. a high amount of ω6 fatty acids (Intralipid^®^) emulsions on the endothelial barrier function. EA.hy926 cell line was cultured and incubated with 0.01, 0.1, and 1 mM lipid emulsions. The influence of these lipid emulsions on the barrier function was assessed using ECIS technology, immunofluorescent microscopy, viability measurements by flow cytometry, multiplex cytokines analysis, and qRT-PCR. BODIPY staining confirmed the uptake of fatty acids by endothelial cells. ECIS measurements demonstrated that a high concentration of Omegaven^®^ prevents barrier formation and impairs the barrier function by inducing cell detachment. Moreover, the expression of VE-cadherin and F-actin formation showed a reorganization of the cell structure within 2 h of 1 mM Omegaven^®^ addition. Interestingly, the study’s findings contradict previous studies and revealed that Omegaven^®^ at high concentration, but not Intralipid, induces cell detachments, impairing endothelial cells’ barrier function. In summary, our studies shed new light on the effect of lipid emulsions on the endothelium.

## 1. Introduction

Total parenteral nutrition (TPN) allows intravenous administration of necessary daily nutrients to millions of patients that are unable to ingest, digest, and absorb nutrients. In addition to providing energy, lipid emulsions are crucial for health and cannot be omitted in TPN formulations due to the body’s inability to synthesize de novo essential fatty acids. Among several intravenous commercially available lipid emulsions, Intralipid^®^ is a soybean oil-based lipid emulsion containing n6 polyunsaturated fatty acids (PUFAs) such as linoleic acid. In contrast, Omegaven^®^ is a fish oil-based n3 lipid emulsion containing eicosapentaenoic acid and docosahexaenoic acid.

Administration of TPN is life-saving in patients; however, they cause side effects due to lipid emulsions. Interestingly, according to Herrera et al. [1], Intralipid^®^ contributes to metabolic stress in endothelial cells but does not influence cell viability [2]. On the other hand, Omegaven^®^ has beneficial effects on programmed cell death but decreases the viability of the cells [2] and is responsible for endothelial dysfunctions in cardiovascular pathology [3,4].

Blood vessels are composed of endothelial cells organized in monolayers attached to the basement membrane by tight junctions and attached to each other by adherens junctions which allow a finely regulated barrier function. Vascular endothelial cadherin (VE-cadherin) is a transmembrane protein. The extracellular domain can link in a homotypic manner to the adjacent cells. These cell–cell interactions regulate the barrier permeability. Notably, the cytoplasmic domain is connected to the actin cytoskeleton through the cadherin–catenin complex. VE-cadherin is also a mechanosensitive protein, and the disruption of the VE-cadherin junction leads to the rearrangement of the actin filament [5,6].

## 2. Materials and Methods

### 2.1. Electrical Cell-Substrate Impedance Sensing (ECIS) Experiment

The 96 w 20 idf ECIS plates were seeded with 20,000 cells per well in 250 µL of media, and the barrier formation was monitored by an ECIS^®^Zθ (Z Theta) device (Applied Biophysics, Troy, NY, USA). Three different layouts were performed during this experiment, (A) with the seeding time point, (B) during the log phase approximately 2 h after seeding, and (C) after the barrier formation. The different lipid emulsions concentrations were added by removing 125 µL media and adding 125 µL of 2 × emulsion with the desired final concentration. Multifrequency mode was used for measuring the resistance, and data were collected for at least 72 h using ECIS software (Applied Biophysics, Troy, NY, USA).

### 2.2. Cell Culture and Immunofluorescence (IF) Staining

Lipid emulsions were purchased from Fresenius Kabi (Bad Homburg, Germany). Volumes of 100 mM Omegaven^®^ and 200 mM Intralipid^®^ were diluted with the medium to obtain final concentrations of 0.01 mM, 0.1 mM, and 1 mM.

Cells were cultured at 37 °C in a humidified 5% CO2 incubator in DMEM medium (Gibco, Thermo Fisher Scientific, Waltham, MA, USA) supplemented with 10% Fetal Bovine Serum (FCS) (PAN Biotech, Aidenbach, Germany) and 1% Sodium Pyruvate (Thermo Fisher Scientific, Massachusetts, United states) according to the protocol provided by the distributor (ATCC CRL 2922). HY926 cells were seeded on glass coverslips prior to treatment with Omegaven^®^ or Intralipid^®^ at different concentrations (0.01, 0.1, and 1 mM). When cells reached full confluency, different concentrations were added and incubated for two hours with the cells. After incubation, cells were fixed using 4% paraformaldehyde for 20 min, washed three times with PBS, and immersed with blocking buffer (PBS pH 7.2, 5% BSA) for at least 1 h at room temperature. After blocking, slides were incubated with primary antibodies diluted in blocking at 4 °C overnight. The next day, secondary antibodies were added for 1 h. Finally, coverslips were mounted on the glass slides with the ProLong™ Gold Antifade Mountant with DAPI (P36935-Thermo Fisher Scientific). Anti-human F-actin (ab130935) and anti-human VE-Cadherin (ab33168) (Abcam, Cambridge, United Kingdom) antibodies were used in the immunofluorescence staining. Images were collected using a ZEISS Axio Imager Z2 microscope equipped with a ZEISS mono and color Axiocam 503 and a ZEISS ApoTome.2 for optical sectioning. To quantify neutral lipid content by fluorescence microscopy 4,4-difluoro-1,3,5,7,8-pentamethyl-4-bora-3a,4a-diaza-s-indacene (BODIPY 493/503) dye was used as previously described [7].

### 2.3. Multiplex Cytokines Analysis

Cytokines concentration in cell culture supernatants was measured using a Bio-Plex Pro Human Cytokine 17-plex assay (#M5000031YV, Bio-Rad Laboratory, Hercules, CA, USA) according to the pre-optimized protocol based on the methodology provided by the manufacturer. Assay #M500031YV was used, which detects: G-CSF, IL-4, IL-10, MIP-1beta, GM-CSF, IL-5, IL-12 (p70), TNF-alpha, IFN-gamma, IL-6, IL-13, IL-1beta, IL-7, IL-17A, IL-8, IL-2, MCP-1. Data were collected and analyzed using a Bio-Rad BioPlex 200 instrument equipped with Bio-Plex Manager software version 6.0 (Bio-Rad Laboratory, Hercules, CA, USA).

### 2.4. Statistical Analysis

All data are presented as means ± standard deviation (S.D.). Statistical analysis was conducted using GraphPad Prism 8 software (GraphPad, San Diego, CA, USA). Statistical comparisons between groups were performed using one-way ANOVA followed by Tukey post-hoc. *p* values below 0.05 were considered significant. Two-way ANOVA was used, where each row represents a different time point, and matched values are stacked into a subcolumn with multiple comparisons. Within each row, columns are compared (simple effects within rows). Each cell mean is compared with the control cell mean on that row (control = unstimulated). *p* values below 0.05 were considered significant.

## 3. Results

**Figure 1:**
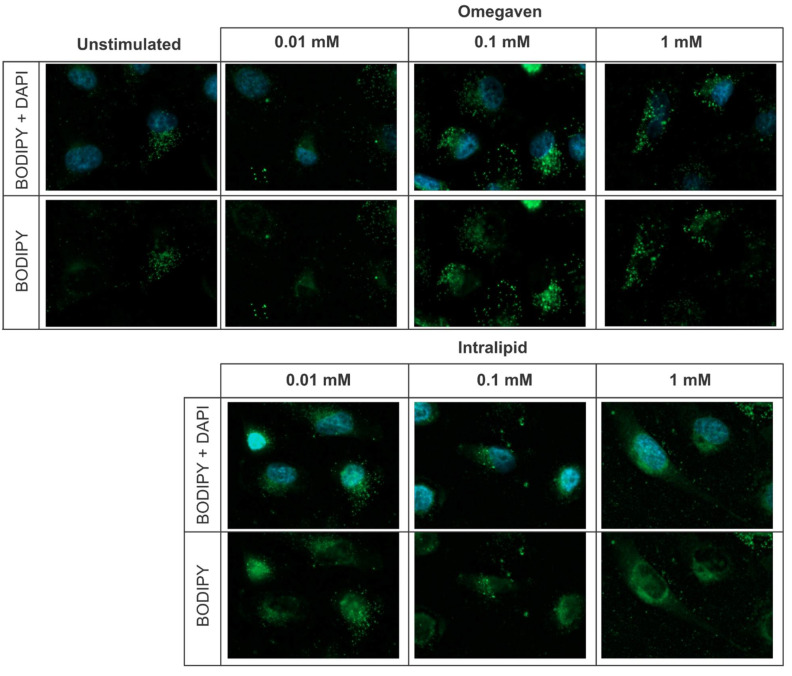
Endothelial cells uptake Omegaven^®^ and Intralipid^®^ emulsions. Endothelial cells were stimulated with 0.01, 0.1, or 1 mM Omegaven^®^ or Intralipid^®^ emulsions for 24 h, and intracellular lipid droplet uptake were measured by BODIPY 493/503 dye staining.

**Figure 2:**
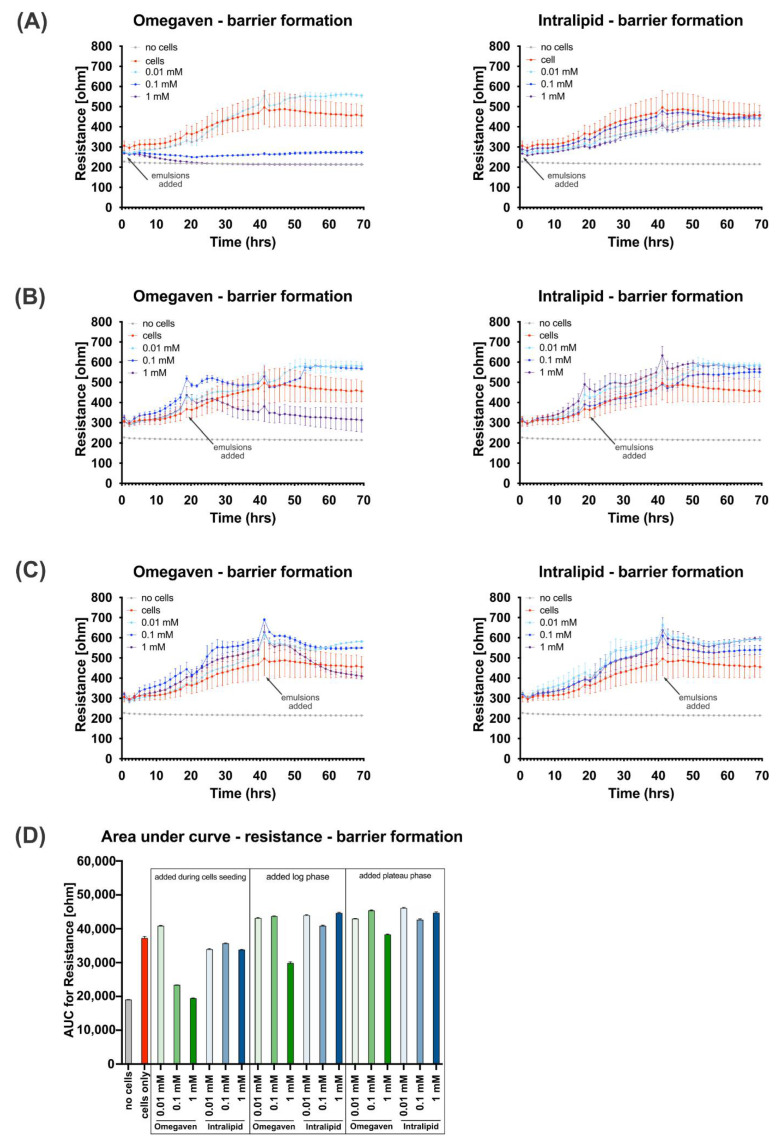
Omegaven^®^ reduces the barrier formation at high concentrations. Omegaven^®^ and Intralipid^®^ lipid emulsions at 0.01, 0.1 and 1 mM concentrations were added to endothelial cells during cell seeding on the ECIS electrode (**A**), during the logarithmic growth phase (**B**), and in cells of the plateau growth phase (**C**). The barrier formation was monitored by an ECIS^®^Zθ (Z Theta) device, and 60 measurements of resistance (ohm) in 70 h were recorded for cells grown in triplicates. In addition, areas under the curve (**D**) for the graph presented in (**A**–**C**) were analyzed using (GraphPad, San Diego, CA, USA), X.Y. analysis/Area under curve function.

This study aimed to determine the in vitro effects of Intralipid^®^ and Omegaven^®^ on the endothelial barrier function. In all in vitro experiments, EA.hy926 cells were used. This cell line is an immortalized human endothelial cell line derived by fusing HUVEC with A549 cells. The lipid emulsions uptake was investigated with BODIPY 493/503 dye staining and revealed that both emulsions, Omegaven^®^ and Intralipid^®^, are taken up by endothelial cells (Figure 1). To measure the impact of lipid emulsions on the endothelial barrier permeability, cells were cultured on gold electrodes of the ECIS technology. Three concentrations (1 mM, 0.1 mM, and 0.01 mM) of Omegaven^®^ or Intralipid^®^ were added during cell seeding (Figure 2A), growth phase (Figure 2B), and growth plateau phase (Figure 2C). The ECIS measurements that monitor endothelial barrier formation revealed no significant variation in the cells’ monolayer resistance after Intralipid^®^ addition. However, the highest dose of Omegaven^®^ (1 mM) decreased the resistance of the endothelial cells, indicating impairment of the endothelial barrier formation (Figure 2D).

**Figure 3:**
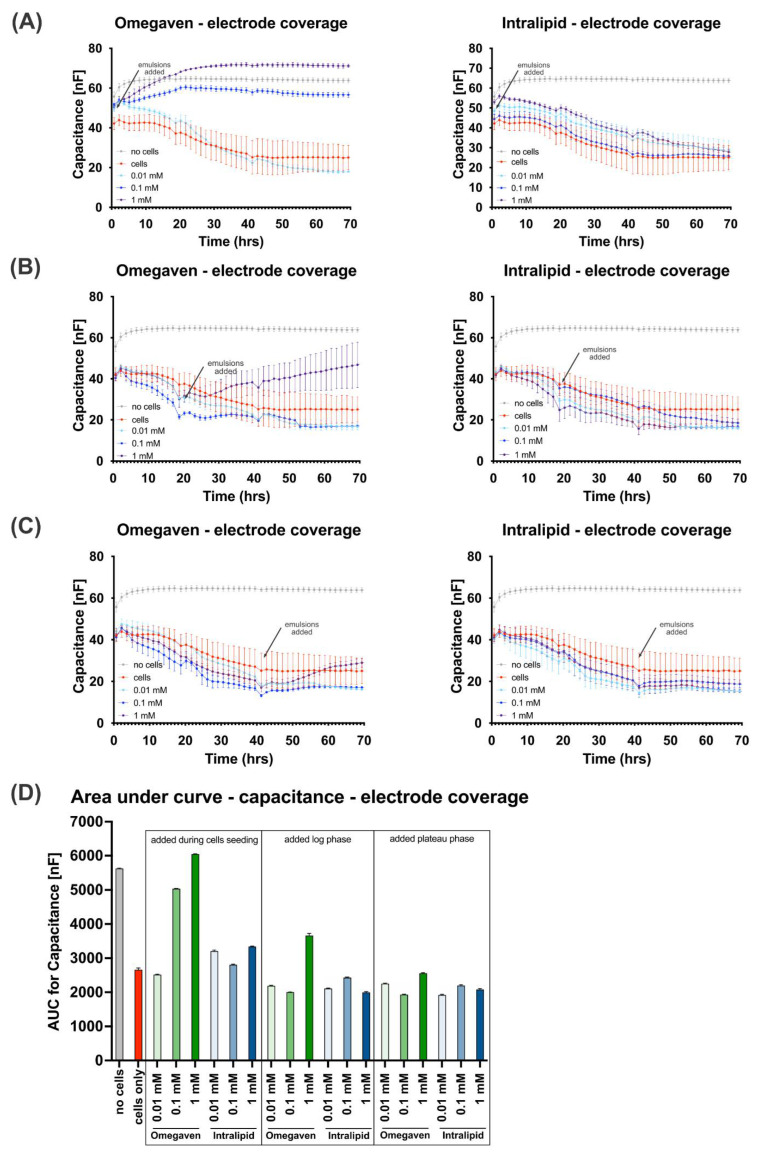
Omegaven^®^ increases the capacitance indicating reduced electrode coverage. Omegaven^®^ and Intralipid^®^ lipid emulsions at 0.01, 0.1 and 1 mM concentrations were added to endothelial cells during cell seeding on the ECIS electrode (**A**), in the cells logarithmic growth phase (**B**), and in cells plateau growth phase (**C**). The electrode coverage was monitored by an ECIS^®^Zθ (Z Theta) device, and 60 measurements of capacitance (nF) in 70 h were recorded for cells grown in triplicates. The decrease in capacitance of cells grown on the electrode is directly proportional to the electrode coverage. Therefore, the higher values indicate the lower electrode coverage by cells. For the graph presented in (**A**–**C**), areas under the curve (**D**) were analyzed using (GraphPad, San Diego, CA, USA), X.Y. analysis/Area under curve function.

Furthermore, the effect of Omegaven^®^ on the endothelial cells was more pronounced when the emulsion was added during cell seeding (Figure 2A). The capacitance measurements that reflect the electrode coverage (Figure 3A–D) confirmed the resistance measurements. Omegaven^®^ added at higher concentrations to cells increased the electrode capacitance and caused low electrode coverage.

In another set of experiments, cells stimulated with the emulsions for 24 h and subsequently left unstimulated in a fresh medium for another 24 h remained attached to culture plates in the presence of all Intralipid^®^ concentrations (0.01, 0.1, 1 mM) as well as two Omegaven^®^ concentrations (0.01, 0.1 mM). However, the highest Omegaven^®^ concentration (1 mM) induced cells detachment from the plastic (Supplementary Figure S1A). This suggests that cells might undergo apoptosis and eventually die. The percentage of cells positive for Annexin V (apoptotic marker) and 7AAD (viability marker) were measured, but no differences in the percentages of necrotic and apoptotic cells were detected 6 h after stimulation with the lipid emulsions (Supplementary Figure S1B).

**Figure 4:**
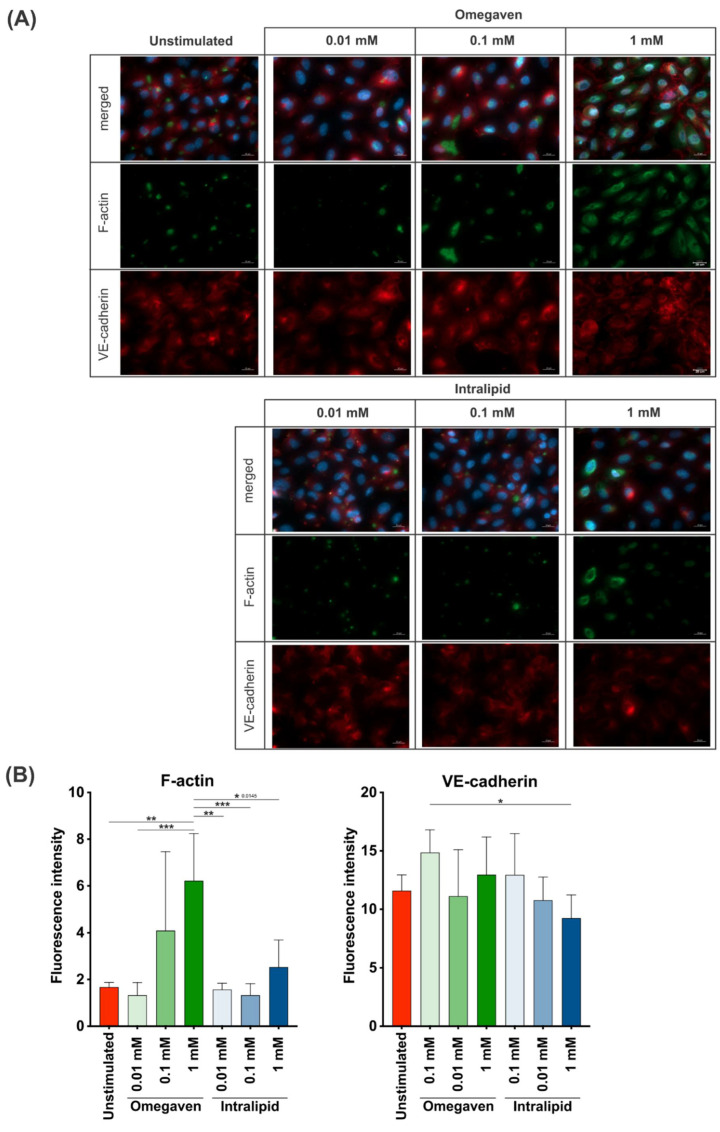
Omegaven^®^ at high doses but not Intralipid^®^ increases the F-acting formation and induces the formation of stress fibers in endothelial cells. Omegaven^®^ and Intralipid^®^ lipid emulsions at 0.01, 0.1 and 1 mM concentrations were added to confluent endothelial cells for 2 h. Cells were stained with anti-VE-cadherin (red) and anti-F-actin (green) antibodies (**A**). Images were acquired at 40× magnification (scale bar 20 um). Fluorescence intensity for F-actin and VE-cadherin staining (**B**) was measured with ImageJ (National Institutes of Health, Stapleton, NY, USA). Five random images acquired for each experimental condition at 40× magnification were used for quantification. (* *p* ≤ 0.05, ** *p* ≤ 0.01, *** *p* ≤ 0.001).

To investigate the effects of lipid emulsions on the confluent endothelial cell cultures, Omegaven^®^ or Intralipid^®^ at 0.01, 0.1 and 1 mM concentrations were added for 2 h, and fluorescence staining of F-actin and VE-cadherin in endothelial cells was performed. The images were acquired at 40× (Figure 4A) and 20× (Supplementary Figures S2A–D and S3A–C) magnification. The immunofluorescence staining revealed a low F-actin formation in unstimulated cells and cells stimulated with the lower Intralipid^®^ concentrations (0.01, 0.1 mM), whereas at 1 mM concentration, increased formation of F-actin was observed. On the other hand, incubation of the endothelial cells with Omegaven^®^ increased the formation of F-actin in a dose-dependent manner (Figure 4B and Supplementary Figure S3D). Furthermore, in almost all experimental conditions, VE-cadherin expression was similar to unstimulated cells with continuous staining at the cell-to-cell border. However, the addition of 1 mM of Omegaven^®^ to cells resulted in a membrane reorganization with perpendicular stretching of the VE-cadherin protein (Figure 4A and Supplementary Figure S2D). Furthermore, the fluorescence intensity measurements indicated a dose-dependent increase in the F-actin signal when treated with Omegaven^®^ without changes in VE-cadherin fluorescence intensity (Figure 4B and Supplementary Figure S3D).

## 4. Discussion

Presented results indicate that lipid emulsions containing a high amount of n3 fatty acids (Omegaven^®^) but not n6 fatty acids (Intralipid^®^) impair the endothelial barrier function, which is in contrary to existing evidence indicating that n3 PUFAs improve endothelial function [3,4]. At the same time, due to its high content of n6 PUFAs, Intralipid^®^ is reported to play an important role in inflammatory processes [8]. Moreover, in the presented work, none of the emulsions affected the viability of the endothelial cells. It was previously reported that fish oil-based lipid emulsion (Omegaven^®^) increased the percentage of apoptotic and necrotic cells, indicating a negative effect on cell viability, while the soybean oil-based lipid emulsion (Intralipid^®^) did not influence cell viability [2].

**Figure 5:**
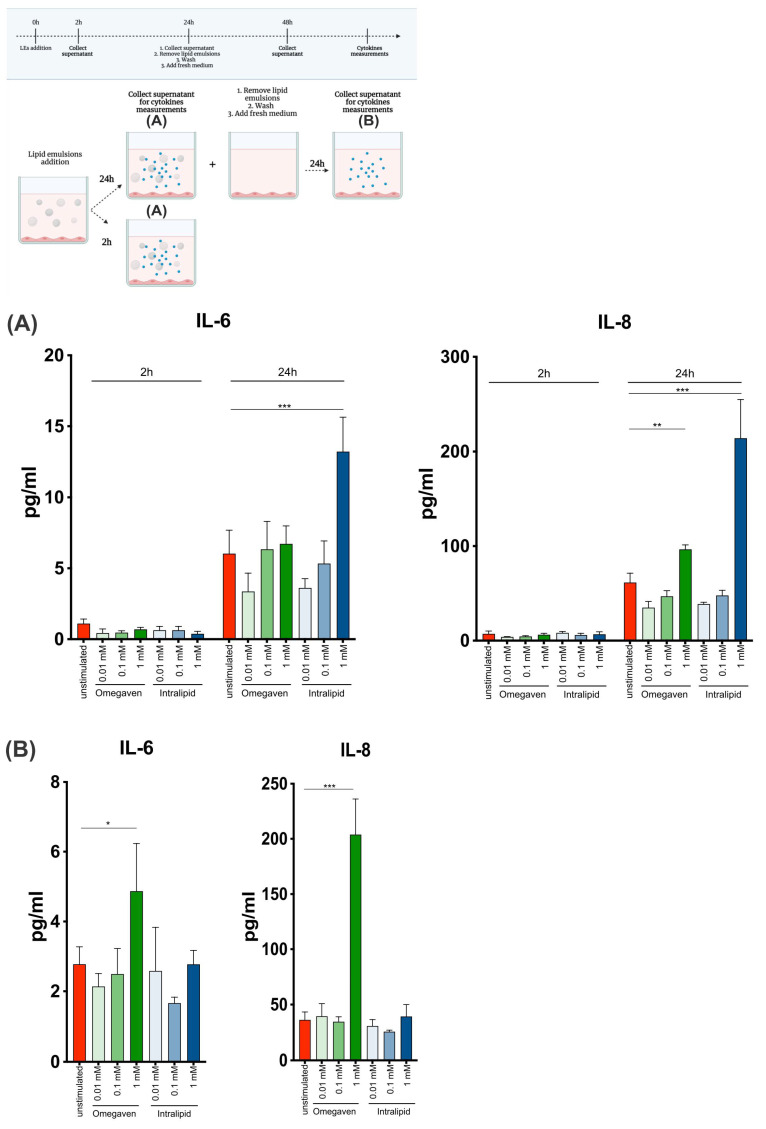
Lipid emulsions induce IL-6 and IL-8 cytokines production from endothelial cells. Endothelial cells were cultured with 0.01, 0.1, 1 mM of Omegaven^®^ or Intralipid^®^, and cell culture supernatant was collected at 2, 24 h (**A**). Next, the same cells were continued to culture in the presence of lipid emulsions for 24 h, lipid emulsions were removed, and fresh medium was added for the next 24 h (**B**). Finally, cytokines levels were measured with a multiplex cytokine assay (n = 3). (* *p* ≤ 0.05, ** *p* ≤ 0.01, *** *p* ≤ 0.001).

Harvey at all [2] showed that Omegaven^®^ and Intralipid® could suppress LPS-induced ICAM-expression. Additionally, Omegaven^®^ suppressed LPS-induced phosphorylation of NF-κB, while Intralipid^®^ did not affect NF-κB phosphorylation. In the presented study, NF-κB activation and phosphorylation were not investigated, but the production of proinflammatory cytokines IL-6 and IL-8 was. The dose and time-dependent increase in the levels of IL-6 and IL-8 were observed upon Intralipid^®^ treatment (Figure 5A). Interestingly, when endothelial cells were cultured with the emulsions for 24 h and left unstimulated for another 24 h, IL-6 and IL-8 production also increased upon 1 mM Omegaven^®^, suggesting prolonged effects of Omegaven^®^ on endothelial cells (Figure 5B).

The highest concentration of Omegaven^®^ induced a reorganization of the cell structure, while Intralipid^®^ does not modify the actin cytoskeleton (Figure 4). F-actin formation significantly increases in dose-dependent manner after Omegaven^®^ addition, indicating stress-induced alterations (Figure 4 and Supplementary Figure S2B–D). Interestingly, it has been previously shown that change in actin cytoskeleton in the form of depolymerization or hyper-polymerization reduces the barrier properties of endothelial cells in vivo and in vitro (MyEnd cell line) [6]. It appears that Omegaven^®^ induces cells activation similar to conditions associated with inflammation.

The addition of 1 mM Omegaven^®^ induced perpendicular staining of VE-cadherin (Figure 4 and Supplementary Figure S2D). In concordance with ECIS results, this suggests that cell–cell junction separation may lead to the endothelial barrier’s disruption. This pattern has been observed previously in EA.hy926 and HUVEC cells in contact with thrombin [9].

In summary, our studies shed new light on the effect of lipid emulsions on the endothelium. Furthermore, as endothelial cells are the first cells that are in contact with lipid emulsions during TPN infusion, our results might explain some of the side effects observed with Omegaven^®^-based TPN. Therefore, primary endothelial cells should be collected from healthy subjects and patients suffering from inflammatory diseases to better understand the effect of different lipid emulsions on the endothelium.

## Data Availability

The data presented in this study are available on request from the corresponding author.

## Supplementary Materials

The following supporting information can be downloaded at: https://www.mdpi.com/article/10.3390/cells11142202/s1, Figure S1: Omegaven and Intralipid emulsions do not affect the viability of endothelial cells, but Omegaven induces cell detachment; Figure S2: Omegaven at high doses but not Intralipid increases F-actin formation; Figure S3. Omegaven but not Intralipid at high doses increases F-actin formation.
