# Supplementary material for: Endothelial Barrier Disruption by Lipid Emulsions Containing a High Amount of N3 Fatty Acids (Omegaven) but Not N6 Fatty Acids (Intralipid)
Source: Cells. 2022 Jul 14;11(14):2202. doi: 10.3390/cells11142202 (PMC9320111; doi:10.3390/cells11142202)
Supplement: Supplementary file 1 [file cells-11-02202-s001.zip › cells-1793892-supplementary.pdf]

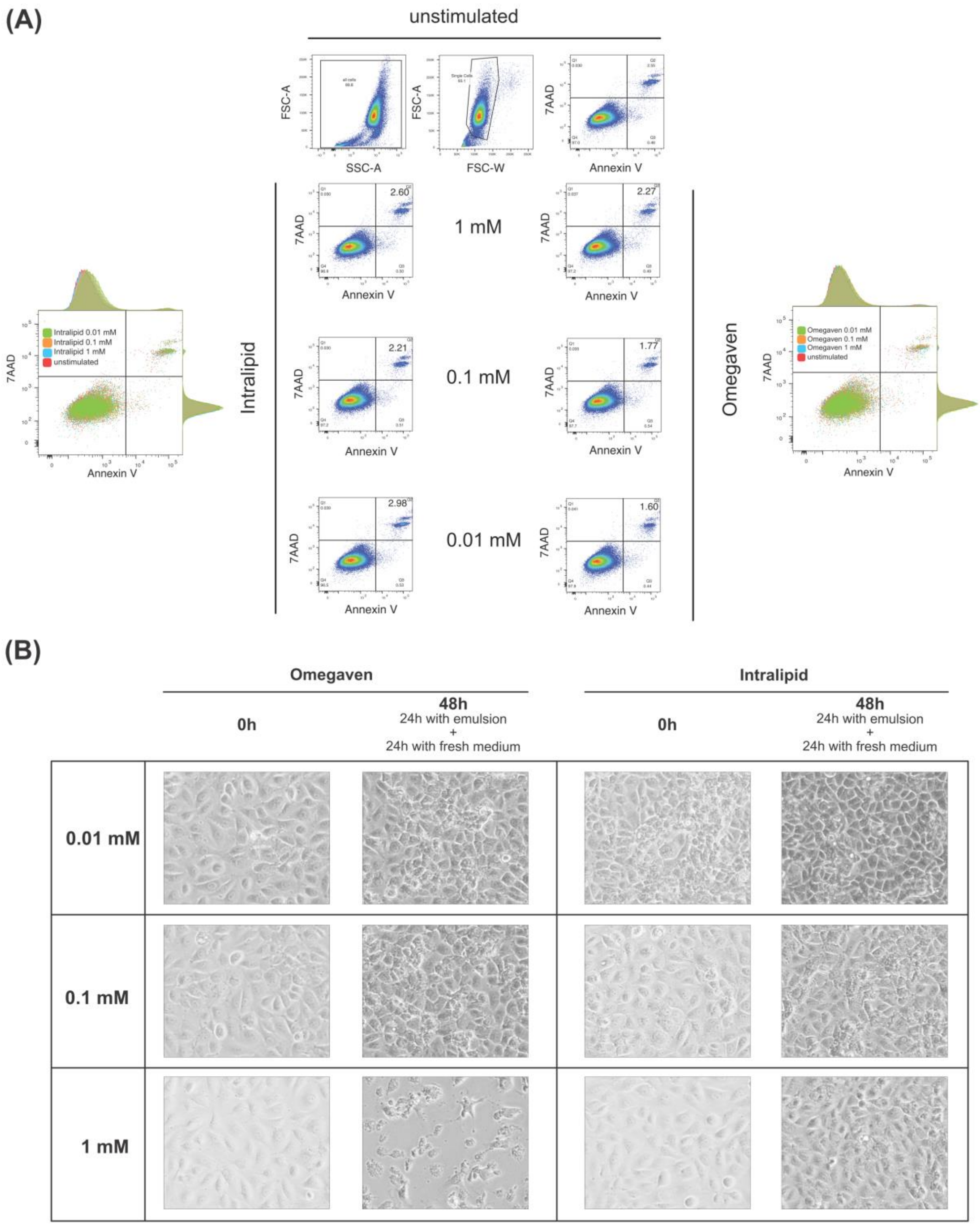

Figure S1. Omegaven and Intralipid emulsions do not affect the viability of endothelial cells, but Omegaven induces cell detachment. Omegaven or Intralipid emulsions were added to endothelial cells cultured in 24 well plates for 24h, next lipid emulsions were removed, and fresh medium was added for another 24h. Cells were imaged with a brightfield microscope before lipids emulsions were added and 48h later. Images acquired at magnification x20 (A). Lipid emulsions were added to endothelial cells for 6h, and 7AAD and Annexin V staining measured cell viability and the induction of apoptosis, respectively. Addition of lipid emulsions did not change the percentage of 7AAD(+) /

Annexin V(-) dead cells, and 7AAD(+) / Annexin V(+) apoptotic cells as compared to unstimulated cells (B).

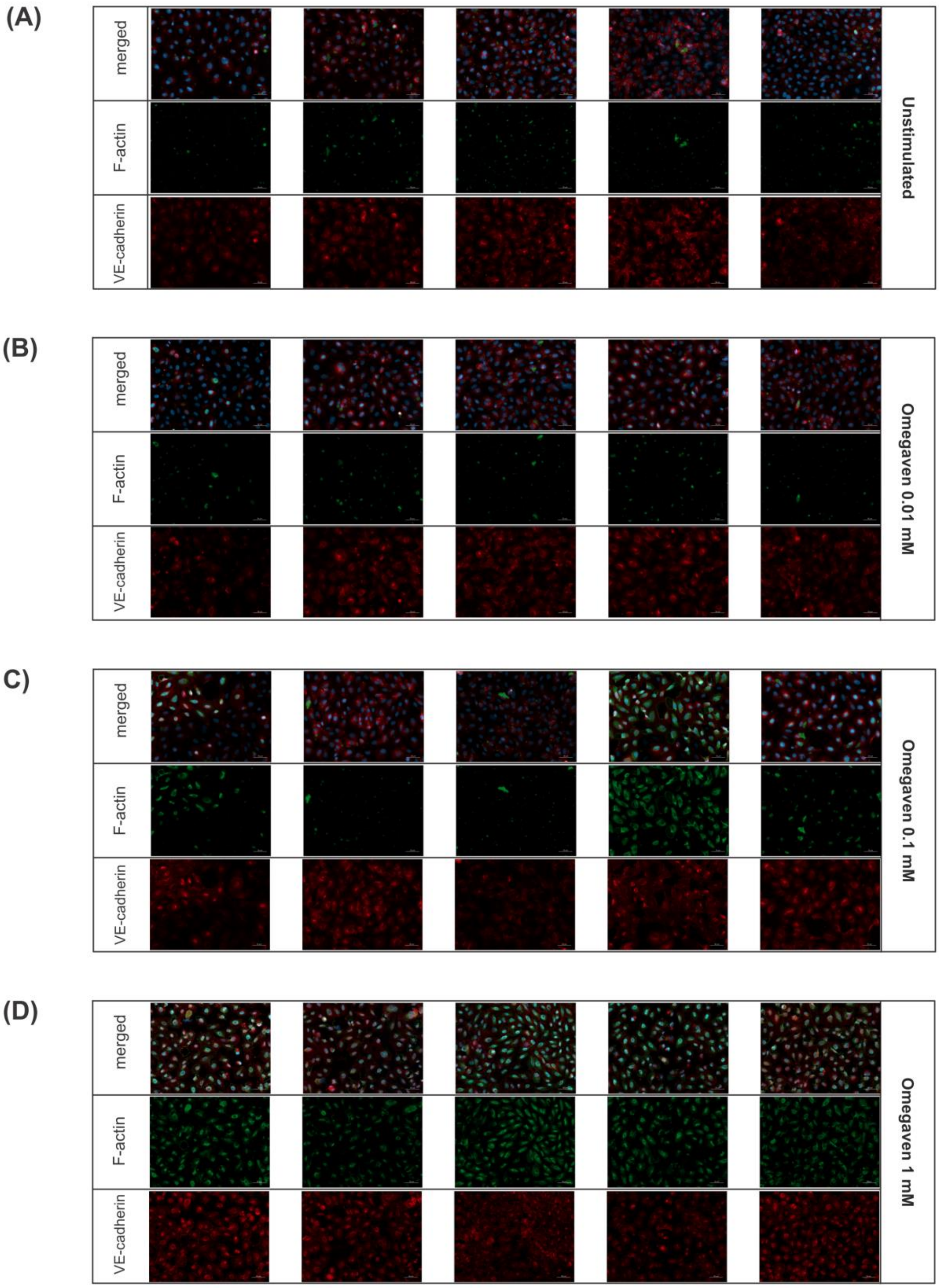

Figure S2. Omegaven at high doses but not Intralipid increases F-actin formation. Figure supplements the main Figure 2 of the manuscript. Five random images for every experimental condition at 20x magnification are included: unstimulated cells (A), cells stimulated with 0.01mM (B), 0.1mM (C) and 1mM of Omegaven (D).

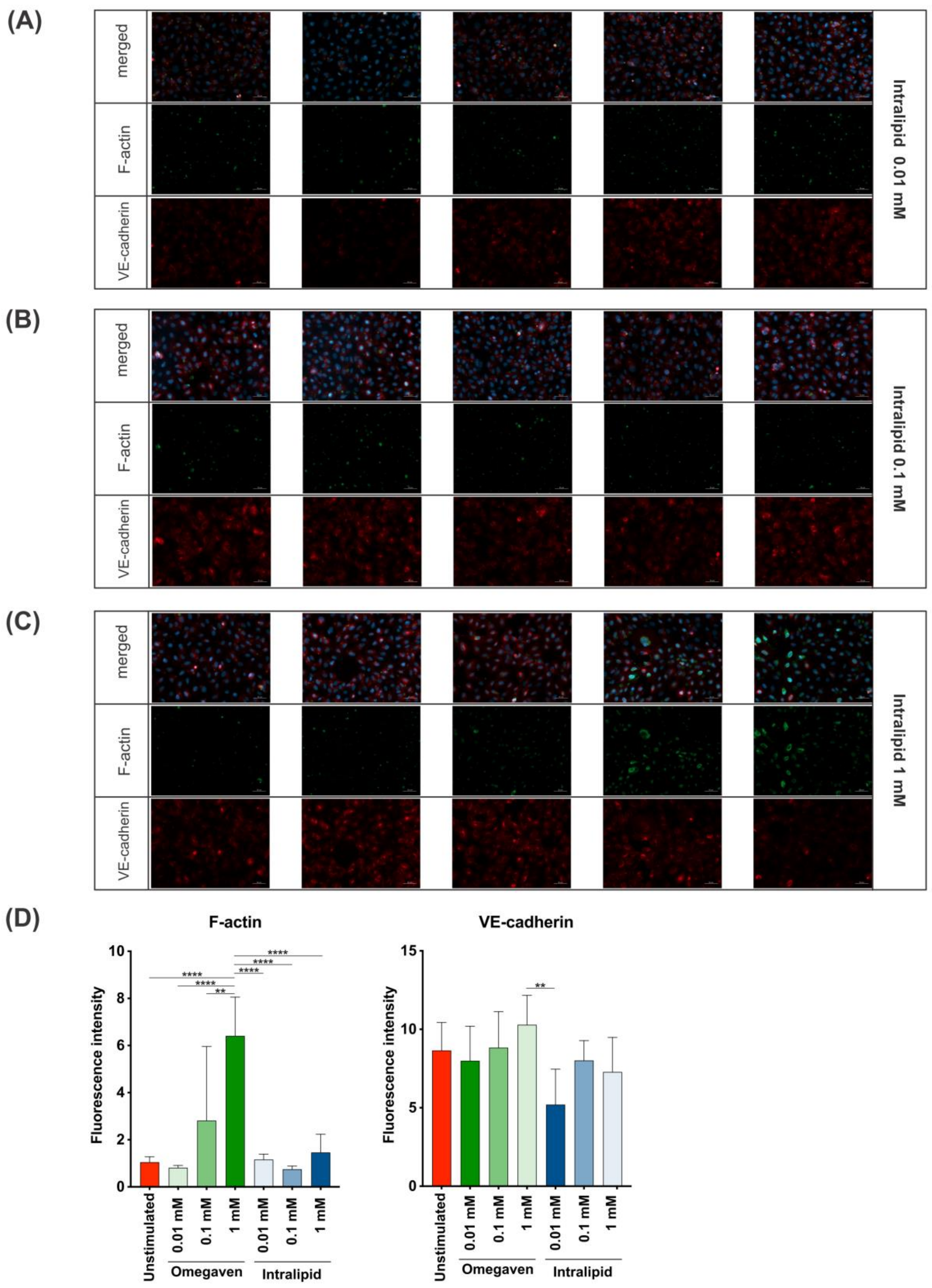

Figure S3. Omegaven but not Intralipid at high doses increases F-actin formation. Figure supplements the main Figure 2 of the manuscript and is a continuation of Supplementary Figure 2. Five random images for every experimental condition at 20x magnification are included: cells stimulated with 0.01mM (B), 0.1mM (C) and 1mM of Intralipid (D). Fluorescence intensity for F-actin and VE-cadherin staining (D) was measured with ImageJ. Five images acquired for every experimental condition at 20x magnification were used for quantification.
